# Comparative efficacy and acceptability of pharmacotherapies for postpartum depression: A systematic review and network meta-analysis

**DOI:** 10.3389/fphar.2022.950004

**Published:** 2022-11-24

**Authors:** Qing Zhang, Xiaoli Dai, Wei Li

**Affiliations:** ^1^ Department of Clinical, Jiangsu Vocational College of Medicine, Yancheng, China; ^2^ Department of Medical Imaging, Jiangsu Vocational College of Medicine, Yancheng, China

**Keywords:** antidepressant, postpartum depression, network meta-analysis, pharmacotherapy, randomized controlled trial

## Abstract

**Purpose:** To evaluate the efficacy and tolerability of pharmacotherapies for postpartum depression (PPD).

**Method:** We performed a computerized search of MEDLINE (Ovid and PubMed), Embase, Cochrane Library, Web of Science, and Google Scholar to identify eligible randomized controlled trials (RCTs) before 31 March 2022. We calculated standardized mean differences (SMDs) for continuous outcomes and odds ratios (ORs) for dichotomous outcomes with the random-effects model. The tolerability of antidepressants in terms of early dropouts was investigated. The surface under the cumulative ranking curve (SUCRA) was used for ranking the outcomes. Quality assessment of the included studies was performed using the Cochrane Collaboration’s tool.

**Results:** A total of 11 studies with 944 participants were included in this network meta-analysis, involving nine antidepressants. With respect to efficacy, only estradiol and brexanolone were significantly more effective than the placebo (*p* < 0.05), and the calculated SUCRA indicated that estradiol (94.3%) had the highest probability ranking first for reducing the PPD, followed by paroxetine (64.3%) and zuranolone (58.8%). Regarding tolerability, a greater percentage of patients treated with brexanolone experienced early dropout as compared to those treated with most other antidepressants.

**Conclusion:** Only estradiol and brexanolone showed significantly higher efficacy than the placebo. According to the SUCRA ranking, estradiol, paroxetine, and zuranolone were the three best antidepressants. Concerning acceptability in terms of early dropouts, brexanolone was less well-tolerated than other antidepressants.

## Introduction

Postpartum depression is one of the most common complications of childbirth, with an estimated prevalence of 10–20% worldwide (Howard et al., 2014; Ko et al., 2017). According to the American Psychiatric Association’s Diagnostic and Statistical Manual of Mental Disorders, Fifth Edition (DSM-5), PPD is a major depressive episode “with peripartum onset” and is defined as “the onset of mood symptoms occurring during pregnancy or in 4 weeks following delivery” (American Psychiatric Association undefined, 2013). Nevertheless, PPD is variably defined in clinical practice, occurring from four weeks to 12 months after childbirth (Stewart and Vigod, 2016). Although more than half of the women are suffering from PPD, most of them are underdiagnosed and undertreated and the condition can persist for years (Vliegen et al., 2014; Netsi et al., 2018). Some patients with PPD could be cured spontaneously within weeks; however, it is estimated that 20% of the women with this disorder remain to suffer from depression during the first year and 13% after two years (Stewart and Vigod, 2016). The common symptoms of PPD include sleep disturbance, anxiety, irritability, and a feeling of being overwhelmed as well as an obsessional preoccupation with the baby’s health and feeding (Wisner et al., 2013; Stewart and Vigod, 2016). These severe psychiatric disorders that onset in the immediate postpartum period are called postpartum psychosis, which is rare, has an estimated prevalence of 1–2 cases per 1,000 births, and is often a manifestation of bipolar disorder (Meltzer-Brody et al., 2018a). PPD leads to maternal impaired emotions, loss of work, and negative effects on infant development (Pearson et al., 2013; Verkuijl et al., 2014; Valla et al., 2016) and is associated with an increased risk of both suicide and infanticide (Bergink et al., 2016). Despite the mechanism of PPD being not clearly understood, studies demonstrated that one of the strongest risk factors is previous mood and anxiety problems*,* especially depression during the pregnancy period (Wisner et al., 2013). Some evidence showed that the hypothalamic–pituitary–adrenal (HPA) axis and γ-aminobutyric acid (GABA) signaling may play a role in the pathophysiology of postpartum depression (Meltzer-Brody et al., 2018a). In addition, the rapid decline in plasma concentrations of allopregnanolone, which is a potent positive allosteric modulator of synaptic and extra-synaptic GABA type A (GABA_A_) receptors, demonstrates a relationship between peripartum hormonal fluctuations and GABA regulation (Maguire and Mody, 2008; Mody and Maguire, 2011).

The first-line antidepressants for PPD treatment are selective serotonin reuptake inhibitors (SSRIs); however, some studies reported that the ability of SSRIs in treating and preventing postpartum depression is limited. In recent years, several new antidepressants are developed for treating PPD and showed promising efficacy and acceptability (Kanes et al., 2017; Deligiannidis et al., 2021). In this study, we aimed to compare the efficacy and acceptability of currently available pharmacological treatment for PPD in women.

## Materials and methods

This systematic review and meta-analysis was performed and reported in compliance with the Preferred Reporting Items for Systematic Reviews and Meta-analyses (PRISMA) extension statement for network meta-analyses (Hutton et al., 2015). A research question was established based on the patient index test comparator outcome study (PICOS) design criteria as follows: what are the differences between currently available antidepressant drugs for the treatment of women with PPD? Our goal was to compare these antidepressant drugs, for continuous outcomes we calculated the SMD and for dichotomous outcomes we calculated the OR. The primary outcome of this study was changes from baseline in a depression scale between different antidepressant drugs, which were measured using the 17-item Hamilton rating scale for depression (HAMD-17) score (Hamilton, 1960). The secondary outcomes include clinical remission rate (HAMD-17 score≤7) and response rate (≥50% reduction of score from baseline), as well as acceptability (treatment dropout measured by the proportion of patients who drop out prematurely).

### Search strategy

A systematic search of MEDLINE (Ovid and PubMed), Embase, Cochrane Library, Web of Science, and Google Scholar was performed to identify eligible studies before 30 April 2022, with no language restriction. We included double-blind, randomized controlled trials comparing antidepressants with the placebo or another active antidepressant for the treatment of PPD. Studies using psychotherapy also would be included if it was applied to all intervention groups without difference. The full reports of studies published in peer-reviewed journals were identified. Additionally, we retrieved the ClinicalTrials.gov and the World Health Organization International Clinical Trials Registry Platform for ongoing and completed clinical trials with results (ClinicalTrials, 2021; WHO, 2021). The references listed in eligible articles and reviews were inspected to expand the scope of the searches. The combinations of terms used for the literature search are as follows: {[(“antidepressant” OR “antidepressive”) AND (“agent*” OR “medication*” OR “drug*“)]} OR {(“nortriptyline” OR “estradiol” OR “paroxetine” OR “sertraline” OR “fluoxetine” OR “brexanolone” OR “saffron” OR “zuranolone” OR “SSRI” OR “serotonin reuptake inhibitors” OR “SSRI” OR “serotonin norepinephrine reuptake inhibitor” OR “SNRI”) AND [(“PPD” OR “postnatal” OR “postpartum”) AND (“depression” OR “disorder”)]}.

### Inclusion criteria

We included studies that met all of the following criteria: 1) randomized controlled parallel-group trials involving more than 10 patients, 2) studies aimed to investigate the efficacy and tolerability of antidepressant agents for the treatment of women diagnosed with PPD according to standard operationalized diagnostic criteria (Feighner Criteria, Research Diagnostic Criteria, DSM-III, DSM-III-R, DSM-IV, DSM-5, or ICD-10), 3) with detailed data on changes in the severity of depression syndrome measured with the HAMD-17 score or the Montgomery–Åsberg depression rating scale (MADRS) to assess the efficacy (Montgomery and Åsberg, 1979), and 4) must be original full-length articles.

### Exclusion criteria

We excluded studies if they satisfy any of the following criteria: 1) studies that do not use pharmacotherapy but psychotherapy or other treatment, 2) not reported detailed data to assess the efficacy and tolerability, and 3) conference abstracts, guidelines, editorials, letters, and reviews.

### Data extraction and quality assessment

We used a standardized protocol to extract the following relevant data and results from the included studies: authors, the trial conducted countries, year of publication, the sample size of each treatment group, patient age, dosage, follow-up period, outcome measurement, and corresponding data. The Cochrane Collaboration’s risk of bias tool was used to assess the risk of bias for the included studies (Higgins et al., 2011), and the following seven domains were evaluated as low, high, or unclear risk of bias for individual studies: 1) random sequence generation, 2) allocation sequence concealment, 3) blinding of participants and personnel, 4) blinding of outcome assessment, 5) completeness of outcome data, 6) selective reporting, and 7) other sources of bias. Data extraction and quality assessment were performed by two reviewers (Z.Q. and D.X.L.) independently, and disagreements were resolved through discussion or arbitration by the third reviewer (L.W.).

### Data synthesis and analysis

We conducted a network meta-analysis using the frequentist model to compare the efficacy and tolerability between antidepressants of interest in all RCTs. For individual studies, we calculated the SMD and corresponding 95% confidence intervals (95% CIs) for continuous outcomes at each follow-up time point, whereas for dichotomous outcomes of clinical response and remission rate as well as dropout rate, we calculated the OR and corresponding 95% CIs using a random-effects network meta-analysis model. We created the network plots to visually present the comparison between different treatments, in which the node sizes correspond to study participants and connection widths correspond to the number of studies. We performed a network meta-analysis of the comparative effectiveness or tolerability using the contrast-based network meta-analysis methods. The inconsistency assumption was used to determine the level of disagreement between direct and indirect evidence, which was evaluated using the overall inconsistency test by fitting design-by-treatment in the inconsistency model. In the case of different intervention doses in a single RCT, we combined them into a single intervention group. The SUCRA and the mean ranks were used to rate the treatments, which represented the probability of a given treatment being the best (or worst) option (Salanti et al., 2011). For SUCRA values, 0% indicates that no chance the treatment is the most efficacious, and 100% indicates that the treatment is certainly the most efficacious. As there was no concrete methodology to evaluate the publication bias between studies in network meta-analysis, we used the comparison-adjusted funnel plots to assess the publication bias among treatment comparisons (Chaimani and Salanti, 2012). Comparison-adjusted funnel plots are scatter plots of effect size *versus* precision, in which the substantial asymmetry around the effect estimate suggests the likelihood of publication bias. Heterogeneity across included RCTs was determined with the Cochran ***Q*** test and measured with ***I*** ^2^ statistic: 0%–40%, slight; 30%–60%, moderate; 50%–90%, substantial; and 75%–100%, considerable. All analyses were conducted using Stata version 15.2 (StataCorp, TX, United States), with statistical significance at *p* < 0.05.

## Result

### Literature search

**FIGURE 1:**
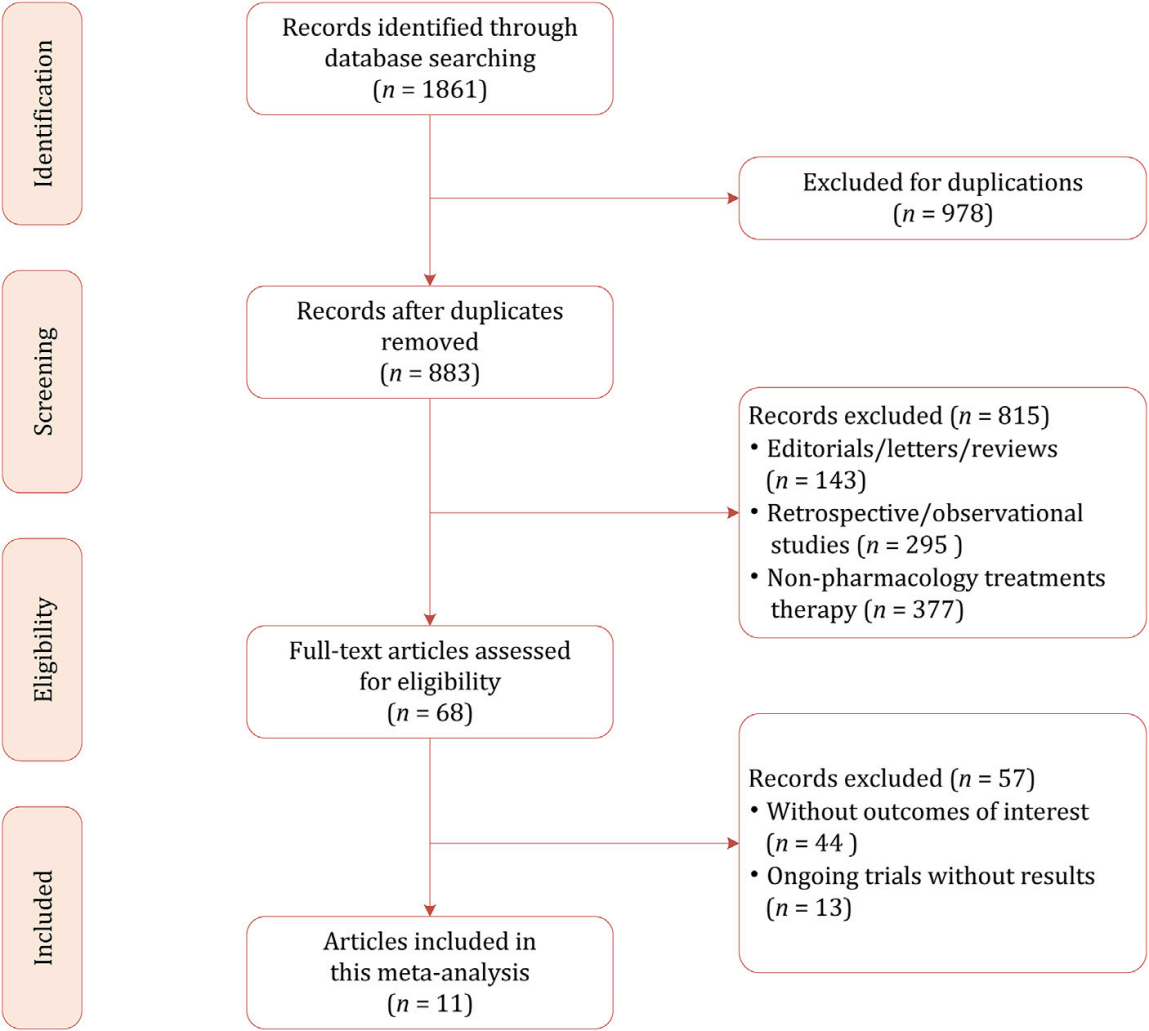
Literature selection process.

Our initial literature search identified 1861 citations, of which 978 were excluded because of duplicates. Among the remaining 883 results, most of them were psychotherapies (i.e., cognitive behavioral therapy and interpersonal therapy) rather than pharmacological therapies (*n* = 437). Other studies excluded were because of the following reasons: for preventing instead of treating PPD (*n* = 51); for the treatment of depression during the prenatal and pregnancy period (*n* = 89); review, systematic review, and meta-analysis (*n* = 196); and retrospective and observational studies (*n* = 42). We conducted the full-text review on the potentially eligible 68 studies, and finally 11 studies including a total of 944 participants were included in this network meta-analysis (Appleby et al., 1997; Wisner et al., 2006; Yonkers et al., 2008; Bloch et al., 2012; O’Hara and McCabe, 2013; Hantsoo et al., 2014; Kanes et al., 2017; Kashani et al., 2017; Meltzer-Brody et al., 2018b; Li et al., 2020; Deligiannidis et al., 2021). The PRISMA flowchart demonstrating the study selection process is presented in Figure 1.

### Study characteristics

Table 1 summarizes the demographic characteristics of the included studies. The study sample ranged from 12 to 150, with a mean age of 25.2–32.1 years. In total, 582 participants were randomly assigned to active antidepressant medications, whereas 362 participants were randomly assigned to the placebo. In seven RCTs, the patients had moderate-to-severe postpartum depressive disorder, with baseline severity on the HAMD-17 score of 21–29, whereas in the remaining three RCTs patients had lower HAMD-17 scores of 16–18 (Bloch et al., 2012; Kashani et al., 2017; Li et al., 2020). In one trial, however, the HAMD-17 score at baseline was 13.67 because it used the Edinburgh postnatal depression scale (EPDS) for screening participants instead of the HAMD-17 (Appleby et al., 1997)*.* The treatment period for the included studies ranged from 4 to 12 weeks, and in most RCTs the period lasted for eight weeks. In five RCTs, the antidepressant medications were SSRIs, including sertraline, fluoxetine, and paroxetine. For the remaining studies, brexanolone was used in three RCTs (Kanes et al., 2017; Meltzer-Brody et al., 2018b), zuranolone was used in one RCT (Deligiannidis et al., 2021), estradiol was used in one RCT (Li et al., 2020), saffron was used in one RCT (Kashani et al., 2017), and nortriptyline was used in one RCT (Wisner et al., 2006).

**TABLE 1:** Characteristics of included RCTs.

Study	Interventions	Location	Year	Number	Age (mean ± SD)	Baseline (HAMD-17, mean ± SD/median, IQR)	Dosage (per day)	Follow-up (weeks)	Scales
Kanes	Brexanolone/Placebo	Multicenter	2017	10/11	27.4 ± 5.3/28.8 ± 4.6	28.1 (27.0–30.0)/28.8 (26.0–32.0)	30-60 ug/h	30d	HAMD-17/MADRS
Meltzer-Brody-1	Brexanolone/Placebo	USA	2018	92/46	27.3 ± 6.1/27.8 ± 6.0 27/27.0 ± 6.0	29.1 ± 2.7/28.4 ± 2.5/28.6 ± 2.5	30-90 ug/h	30d	HAMD-17/MADRS
Meltzer-Brody-2	Brexanolone/Placebo	USA	2018	54/54	28.4 ± 6.1/27.4 ± 5.9	22.6 ± 1.6/22.7 ± 1.6	30-90 ug/h	30d	HAMD-17/MADRS
Appleby	Fluoxetine/Placebo	UK	1997	43/44	26.1/24.5	14.2 (13.0-15.5) 13.9 (12.5-15.4)	NA	12	HAMD-17/EPDS
O’Hara-Iowa	Sertraline/Placebo	USA	2010	23/20	28.7 ± 5.9/ 28.1 ± 5.4	21.5 ± 4.5/20.2 ± 4.4	25-200 mg	12	HAMD-17
O’Hara-WIH	Sertraline/Placebo	USA	2010	33/33	27.8 ± 5.5/ 26.8 ± 4.9	22.1 ± 5.0/23.2 ± 4.5	25-200 mg	12	HAMD-17
Kashani	Saffron/Fluoxetine	Iran	2017	32/32	29.21 ± 7.69	16.53 ± 1.48	NA	6	HAMD-17
					32.09 ± 4.99	16.65 ± 1.12			
Hantsoo	Sertraline/Placebo	USA	2013	17/19	29.6 ± 4.0/31.7 ± 3.7	20.6 ± 2.8/23.2 ± 3.9	50-200 mg	6	HAMD-17/EPDS
Wisner	Sertraline/Nortriptyline	USA	2006	55/54	NA	NA	25-200 mg/10-150 mg	8	HAMD-17
Yonkers	Paroxetine/Placebo	USA	2008	35/35	26.1 ± 6.5/25.9 ± 6.5	23.6 ± 4.7/24.7 ± 5.0	10-40 mg	8	HAMD-17
Li	Estradiol/Placebo	USA	2020	6/6	30.5 ± 6.2/32.7 ± 5.5	18.2 ± 7.3/18.3 ± 3.4	5 mg	6	HAMD-17/EPDS
Bloch	Sertraline/Placebo	Israel	2012	20/20	NA	18.40 ± 4.83 16.05 ± 4.84	25-100 mg	12	EPDS/MADRS
Deligiannidis	Zuranolone/Placebo	USA	2021	76/74	29.3 ± 5.4/27.4 ± 5.3	28.4 ± 2/28.8 ± 2	30 mg	45d	HAMD-17/MADRS

Abbreviations: EPDS, Edinburgh Postnatal Depression Scale; HAMD-17, 17-item Hamilton Rating Scale for Depression score; IQR, interquartile range; MADRS, Montgomery-Åsberg Depression Rating Scale; NA, not available; SD, standard deviation.

### Quality assessment for included RCTs

**FIGURE 2:**
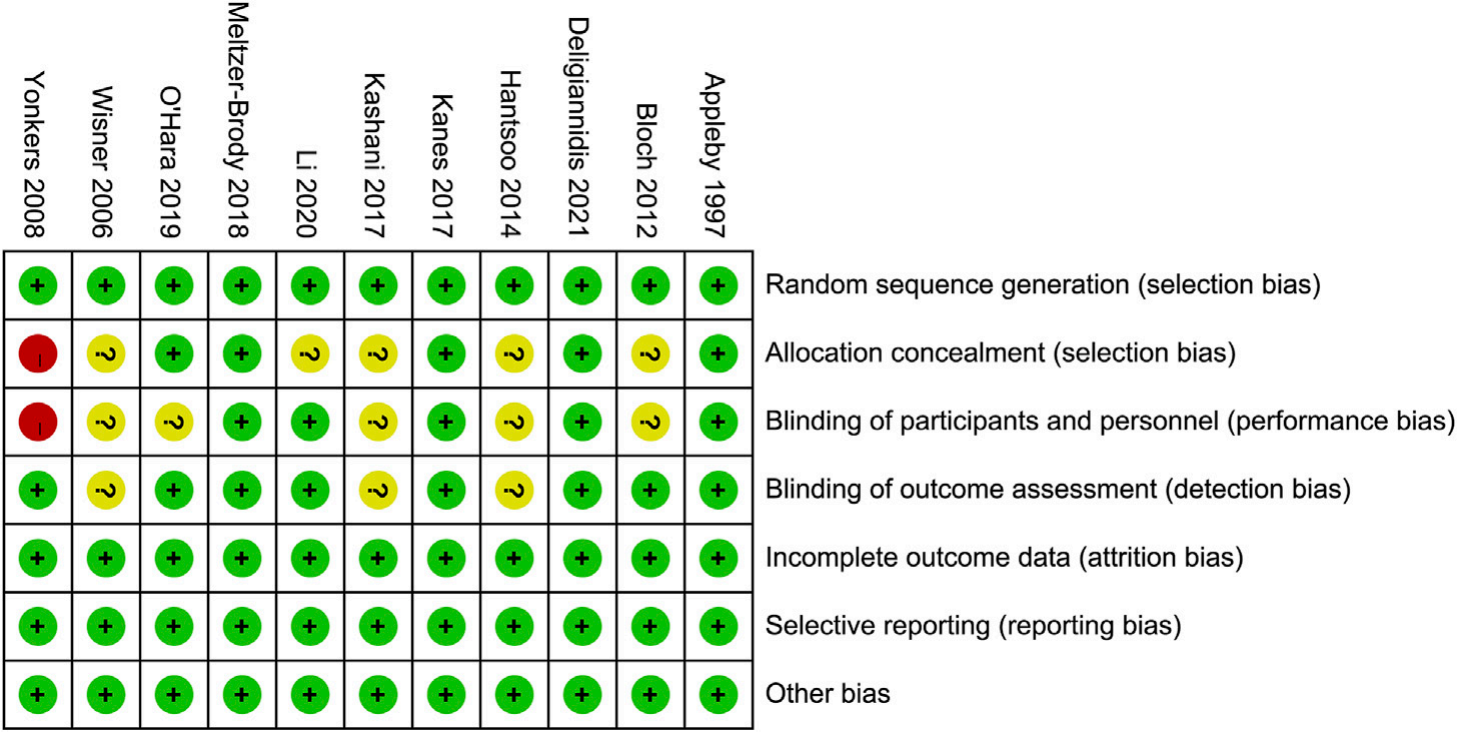
Risk of bias.

**FIGURE 3:**
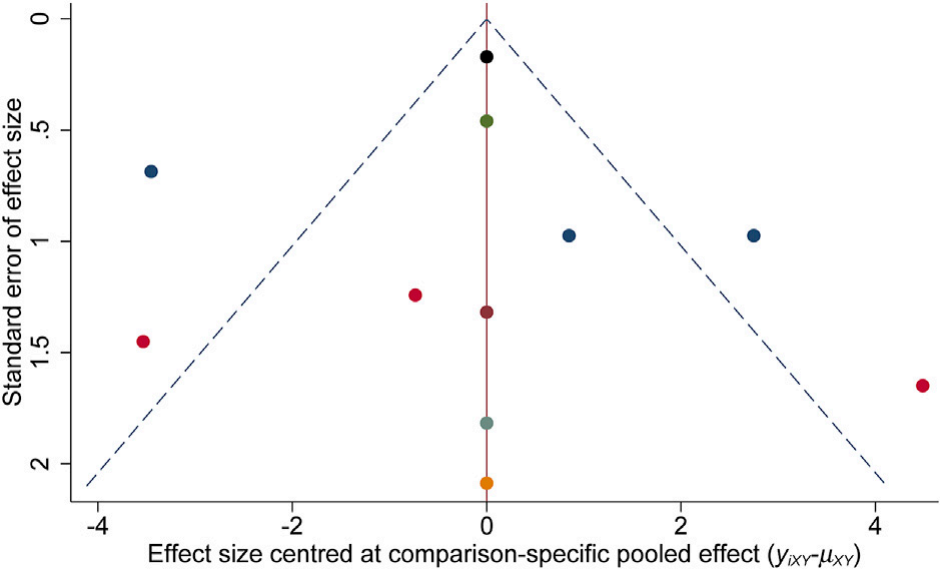
Funnel plots.

Figure 2 demonstrates the risk of bias for included RCTs. Of the 11 RCTs included in the network meta-analysis, four were assigned at low risk of bias (Appleby et al., 1997; Kanes et al., 2017; Meltzer-Brody et al., 2018b; Deligiannidis et al., 2021), and six were considered unclear risk of bias due to attrition bias (Yonkers et al., 2008; Bloch et al., 2012; O’Hara and McCabe, 2013; Hantsoo et al., 2014; Kashani et al., 2017; Li et al., 2020). Only one RCT scored a high risk of bias because the blinding was not explicitly reported (Yonkers et al., 2008). The results of the comparison-adjusted funnel plots showed that there was no evidence of apparent asymmetry, indicating no significant publication bias (Figure 3).

### Comparison of efficacy between antidepressant medications

**FIGURE 4:**
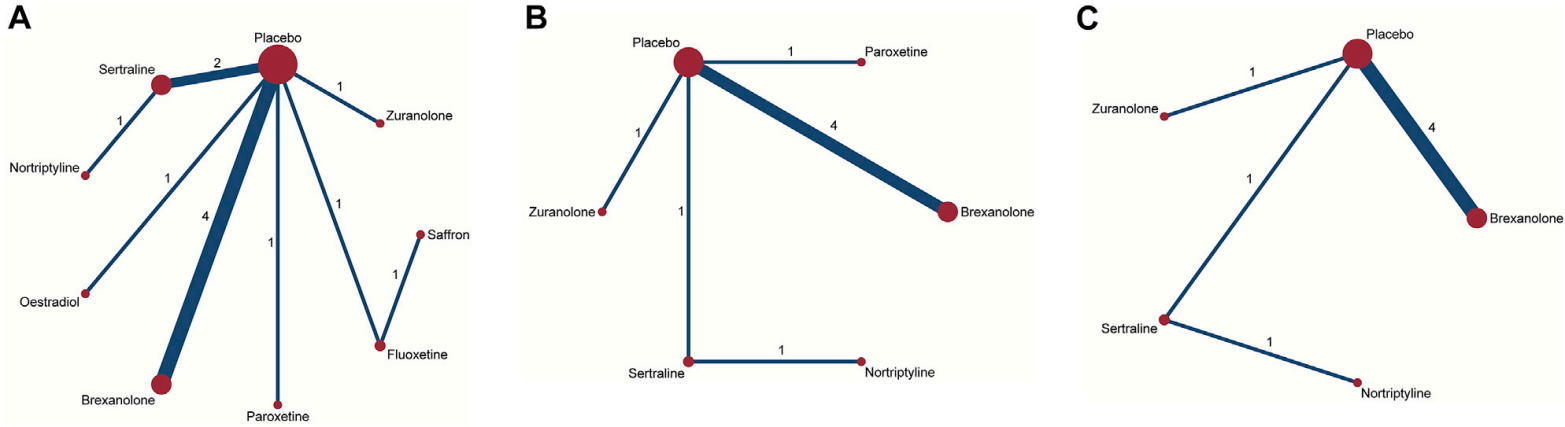
Network plots of treatment comparisons for the efficacy outcomes. Circle size is proportional to the number of study participants assigned to receive each intervention. The line width corresponds to the number of studies comparing the treatments. **(A)** Change in SMD from baseline; **(B)** remission rate; and **(C)** responder rate.

**FIGURE 5:**
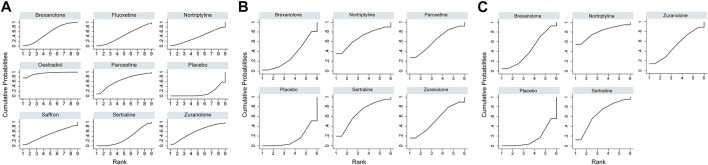
Surface under the cumulative ranking curve probabilities for the ranking. **(A)** Change in SMD from baseline; **(B)** remission rate; and **(C)** responder rate.

For the primary outcome of the mean-changed HAMD-17 score, the network plot consists of nine nodes (Figure 4A), and Table 2 shows the results of the network meta-analysis. As the majority of RCTs were comparisons between antidepressant medications and the placebo, it was unfeasible to check for inconsistency between direct and indirect treatments. There was moderate statistical heterogeneity between studies (***I*** ^2^ = 54.9%) regarding the efficacy of changes in the HAMD-17 score. In all RCTs, the active antidepressant drugs were superior to the placebo for reducing depression, with SMD ranging from −1.70 (95% CI −4.72 to 1.33) to −9.59 (95% CI −15.78 to -3.4). However, our analysis demonstrated that only estradiol and brexanolone were significantly superior to the placebo (*p* < 0.01 and *p* = 0.02, respectively), whereas for all the remaining seven antidepressant medications the results suggested there was no substantial difference between them and the placebo (with *p*-values ranging from 0.08 to 0.54). Regarding the active antidepressant medication, only estradiol was significantly more effective than sertraline, with the SMD of −7.89 (95% CI-14.78 to −1.01), *p* = 0.03. The SUCRA suggested that estradiol (94.3%), paroxetine (64.3%), and zuranolone (58.8%) were superior to other antidepressants in the ranking probability, and the details are presented in Figure 5A.

**TABLE 2:** Network meta-analysis results of the efficacy.

**Oestradiol**	4.89 (-3.62, 13.40)	5.49 (-2.76, 13.74)	6.10 (-0.79, 12.99)	6.78 (-2.67, 16.23)	6.99 (-1.20, 15.18)	7.69 (-0.99, 16.37)	7.89 (1.01, 14.78)	9.59 (3.40, 15.78)
-4.89 (-13.40, 3.62)	**Paroxetine**	0.60 (-7.40, 8.60)	1.21 (-5.38, 7.80)	1.89 (-7.35, 11.13)	2.10 (-5.84, 10.04)	2.80 (-5.64, 11.24)	3.00 (-3.58, 9.58)	4.70 (-1.15, 10.55)
-5.49 (-13.74, 2.76)	-0.60 (-8.60, 7.40)	**Zuranolone**	0.61 (-5.63, 6.86)	1.29 (-7.71, 10.29)	1.50 (-6.16, 9.16)	2.20 (-5.98, 10.38)	2.40 (-3.84, 8.64)	4.10 (-1.36, 9.56)
-6.10 (-12.99, 0.79)	-1.21 (-7.80, 5.38)	-0.61 (-6.86, 5.63)	**Brexanolone**	0.68 (-7.09, 8.44)	0.89 (-5.27, 7.05)	1.59 (-5.21, 8.39)	1.79 (-2.49, 6.06)	3.49 (0.46, 6.52)
-6.78 (-16.23, 2.67)	-1.89 (-11.13, 7.35)	-1.29 (-10.29, 7.71)	-0.68 (-8.44, 7.09)	**Saffron**	0.21 (-4.51, 4.93)	0.91 (-8.48, 10.30)	1.11 (-6.65, 8.87)	2.81 (-4.34, 9.96)
-6.99 (-15.18, 1.20)	-2.10 (-10.04, 5.84)	-1.50 (-9.16, 6.16)	-0.89 (-7.05, 5.27)	-0.21 (-4.93, 4.51)	**Fluoxetine**	0.70 (-7.42, 8.82)	0.90 (-5.26, 7.06)	2.60 (-2.76, 7.96)
-7.69 (-16.37, 0.99)	-2.80 (-11.24, 5.64)	-2.20 (-10.38, 5.98)	-1.59 (-8.39, 5.21)	-0.91 (-10.30, 8.48)	-0.70 (-8.82, 7.42)	**Nortriptyline**	0.20 (-5.09, 5.49)	1.90 (-4.19, 7.99)
**-7.89 (-14.78, -1.01)**	-3.00 (-9.58, 3.58)	-2.40 (-8.64, 3.84)	-1.79 (-6.06, 2.49)	-1.11 (-8.87, 6.65)	-0.90 (-7.06, 5.26)	-0.20 (-5.49, 5.09)	**Sertraline**	1.70 (-1.33, 4.72)
**-9.59 (-15.78, -3.40)**	-4.70 (-10.55, 1.15)	-4.10 (-9.56, 1.36)	**-3.49 (-6.52, -0.46)**	-2.81 (-9.96, 4.34)	-2.60 (-7.96, 2.76)	-1.90 (-7.99, 4.19)	-1.70 (-4.72, 1.33)	**Placebo**

**Note**: Network meta-analysis results of the efficacy in terms of standard mean differences for postpartum depression of different antidepressant medications, which are reported in order of surface under the curve cumulative ranking. Top-ranked treatment listed in the top left corner and rankings proceed down the diagonal. lower than 0 favors the column-defining treatment and in the upper right half, those lower than 0 favors the row-defining treatment. Cells in bold print indicate significant results.

In addition to mean changes from baseline, the remission rate was reported in six studies involving five antidepressant drugs (Wisner et al., 2006; Yonkers et al., 2008; Hantsoo et al., 2014; Kanes et al., 2017; Meltzer-Brody et al., 2018b; Deligiannidis et al., 2021), and the corresponding network plot is shown in Figure 4B. Although no significant differences were found between active antidepressant medications and the placebo (*p* = 0.18–0.48), the SUCRA ranking suggested that nortriptyline (67.9%), sertraline (68.0%), and paroxetine (62.8%) had a higher remission rate than others (Figure 5B). The relative efficacy of the responder rate was reported in five studies, including five antidepressants (Figure 4C) (Wisner et al., 2006; Hantsoo et al., 2014; Kanes et al., 2017; Meltzer-Brody et al., 2018b; Deligiannidis et al., 2021). According to SUCRA, nortriptyline had the greatest likelihood of ranking first (80.0%) and the second was sertraline (64.5%), which is shown in Figure 5C. However, neither of them was significantly superior to the placebo (OR = 6.74, 95% CI 0.46-99.39 and OR = 4.00, 95% CI 0.5-31.83, respectively). No global statistical heterogeneity was noted among studies with respect to both remission (***I*** ^2^ = 0.0%) and responder rates (***I*** ^2^ = 0.0%). In the light of several RCTs that provided the outcomes measured with MADRS, we performed network analysis of efficacy among these RCTs, and the results are presented in Supplementary Table S1 ***.***

### Tolerability and side effects

Regarding comparative acceptability, a total of nine RCTs reported the total number of early dropouts for any reason. No antidepressant medication was found to be significantly inferior to the placebo. Similarly, there was no significant difference between active antidepressants. When treatments were ranked according to SUCRA, brexanolone was less well-tolerated than most other drugs (4.5%), and the following were two SSRIs of fluoxetine (28.6%) and sertraline (29.0%, Supplementary Figure S1). We did not observe statistical heterogeneity between RCTs (I^2^ = 0.0%). As few studies provided details on the most common and serious side effects, it was unfeasible to pool data in terms of this endpoint (Supplementary Table S2). However, we carried out the network meta-analysis in studies providing dropout for side effects and lacking efficacy, and the results were similar to dropout for any reason, which is presented in Supplementary Figure S2.

## Discussion

To the best of our knowledge, this is the first systematic review and network meta-analysis of RCTs of antidepressant medications for women with PPD. While using the changes in the HAMD-17 score from baseline as the endpoint, only estradiol and brexanolone were found to be superior to the placebo after 4–12 weeks of treatment. Among several active antidepressants, we found that only estradiol was significantly more effective than sertraline. The SUCRA indicated that estradiol, paroxetine, and zuranolone were superior to other antidepressant drugs. While using the remission rate as the endpoint of interest, the SUCRA ranking demonstrated that nortriptyline, sertraline, and paroxetine had a higher remission rate than other antidepressants. Again, no significant difference was found among these drugs. In terms of responder rate, despite observing no significant differences in efficacy between treatments through indirect comparisons among studies, the SUCRA indicated that nortriptyline had the greatest likelihood of ranking first. In the study of Cooper et al., they performed an indirect comparison between brexanolone and SSRIs with EPDS and HAMD-17 as the measurements. According to their analyses, brexanolone was more effective within 60 h and can potentially lead to better treatment and symptom reduction for mothers with PPD (Cooper et al., 2019). In our study, the EPDS was not used because of insufficient data; moreover, the EPDS was developed as a screening tool and may not have captured all relevant aspects of changing PPD symptoms.

Concerning the tolerability profile, the SUCRA ranking revealed that brexanolone has a higher dropout rate for any reason than most other antidepressant medications. On the other side, two SSRIs of fluoxetine and sertraline were more well-tolerated than other antidepressants; however, considering that many dropouts were lost to follow-up, the results should be regarded with caution. Because of insufficient data, it was difficult to perform a comparison regarding the dropout rate for the most common and serious side effects. Another problem that should be noted was that brexanolone was administered by injection and not orally, which may bring inconvenience in outpatient settings.

Depending on the severity and functional status of women diagnosed with PPD, the treatment option varied widely. For mild disorder, psychosocial interventions by trained health professionals are recommended as the first-line interventions, whereas for women with moderate disorder, formal psychotherapy over 3–4 months should be considered (Antenatal and postnatal mental health: clinical management and service guidance, 2014; Dennis and Dowswell, 2013). When PPD is severe or cannot be resolved with psychological treatment, antidepressant drugs are required. At present, the majority of trials for the treatment of PPD were focused on non-pharmacological treatments. By comparison, far fewer trials focused on pharmacotherapy for the treatment of PPD, and most of these studies were placebo-controlled trials (Lam et al., 2013). In the current network meta-analysis, we observed moderate heterogeneity, which is commonly seen in psychological treatment research. Nevertheless, because of insufficient data, we were not able to explore potentially important clinical and demographical information at the individual patient level such as age, duration of illness, and especially the severity of symptoms (ranging from 16.5 to 29.1 at baseline with the HAMD-17 scale). Although without these clinical subgroups, results might limit the applicability of our study, and it was intended as a methodological strength to assure transitivity in the network. For non-postpartum populations, there is evidence of some benefits of combined psychotherapy and pharmacotherapy for functional outcomes. Nevertheless, trials of combination therapy for postpartum depression are scarce. Considering that several newer RCTs provided the MADRS scale, we compared different antidepressants in RCTs providing MADRS, and the results are consistent with the measurement of HAMD-17. In light of a few head-to-head comparison studies between different antidepressants, the results of the current network meta-analysis were derived from indirect comparisons of treatments. Compared with pairwise comparison, network meta-analysis can supply a more precise estimate of the relative efficacy and tolerability and allow treatments to be ranked to assist clinical decisions (Salanti et al., 2008; Salanti, 2012). Nevertheless, due to the high risk of bias of included RCTs, the quality of the evidence from this study is low. Therefore, the results of the current network meta-analysis should be regarded with caution. However, in the absence of a direct comparison between antidepressants, our findings represent the best currently available evidence for patients and clinicians to inform first-line and second-line treatment decisions for PPD.

There are some limitations to our study which should be considered. First, in more than half of the RCTs, the antidepressant medications were tested in less than 100 patients, and small sample trials usually result in larger treatment effects. Second, many RCTs were conducted 10 years ago and did not report adequate information about allocation concealment and sequence generation. Furthermore, the endpoints used to judge treatment efficacy were less stringent than those used in earlier RCTs relative to those newer ones. Third, some RCTs reported detailed efficacy at varied time points such as 4, 24, and 48 h; however, it is unfeasible to pool these data because of insufficient details*.*

## Conclusion

All active antidepressant medications demonstrated higher efficacy relative to the placebo while using changes in the HAMD-17 score from baseline as the endpoint; however, only estradiol and brexanolone were significantly superior to the placebo. The SUCRA ranking revealed that estradiol, paroxetine, and zuranolone were superior to other antidepressants. Concerning the acceptability of dropouts, the SUCRA ranking indicated that brexanolone is less well-tolerated than most other antidepressants.

## Data Availability

The original contributions presented in the study are included in the article/Supplementary Material; further inquiries can be directed to the corresponding authors.

## Supplementary material

Supplementary Figure S1 SUCRA of dropout for any reason.

Supplementary Figure S2 SUCRA of dropout for adverse events.

## References

[B1] American Psychiatric Association undefined A. P. (2013). Diagnostic and statistical manual of mental disorders. 5th ed., 5. Arlington: DSM.

[B2] Antenatal and postnatal mental health: clinical management and service guidance (2014). London: National Institute for Health and Care Excellence NICE. http://www.ncbi.nlm.nih.gov/books/NBK553127/ (Accessed March 8, 2022). 31990493

[B3] Appleby L. Warner R. Whitton A. Faragher B. (1997). A controlled study of fluoxetine and cognitive-behavioural counselling in the treatment of postnatal depression. BMJ 314, 932–936. 10.1136/bmj.314.7085.932 9099116 PMC2126383

[B4] Bergink V. Rasgon N. Wisner K. L. (2016). Postpartum psychosis: Madness, mania, and melancholia in motherhood. Am. J. Psychiatry 173, 1179–1188. 10.1176/appi.ajp.2016.16040454 27609245

[B5] Bloch M. Meiboom H. Lorberblatt M. Bluvstein I. Aharonov I. Schreiber S. (2012). The effect of sertraline add-on to brief dynamic psychotherapy for the treatment of postpartum depression: A randomized, double-blind, placebo-controlled study. J. Clin. Psychiatry 73, 235–241. 10.4088/JCP.11m07117 22401479

[B6] Chaimani A. Salanti G. (2012). Using network meta-analysis to evaluate the existence of small-study effects in a network of interventions. Res. Synth. Methods 3, 161–176. 10.1002/jrsm.57 26062088

[B7] ClinicalTrials. . Home - ClinicalTrials.gov. https://clinicaltrials.gov (Accessed June 22, 2021).

[B8] Cooper M. C. Kilvert H. S. Hodgkins P. Roskell N. S. Eldar-Lissai A. (2019). Using matching-adjusted indirect comparisons and network meta-analyses to compare efficacy of brexanolone injection with selective serotonin reuptake inhibitors for treating postpartum depression. CNS Drugs 33, 1039–1052. 10.1007/s40263-019-00672-w 31642037 PMC6825025

[B9] Deligiannidis K. M. Meltzer-Brody S. Gunduz-Bruce H. Doherty J. Jonas J. Li S. (2021). Effect of zuranolone vs placebo in postpartum depression: A randomized clinical trial. JAMA Psychiatry 78, 951–959. 10.1001/jamapsychiatry.2021.1559 34190962 PMC8246337

[B10] Dennis C-L. Dowswell T. (2013). Psychosocial and psychological interventions for preventing postpartum depression. Cochrane Database Syst. Rev., CD001134. 10.1002/14651858.CD001134.pub3 23450532 PMC11936315

[B11] Hamilton M. (1960). A rating scale for depression. J. Neurol. Neurosurg. Psychiatry 23, 56–62. 10.1136/jnnp.23.1.56 14399272 PMC495331

[B12] Hantsoo L. Ward-O’Brien D. Czarkowski K. A. Gueorguieva R. Price L. H. Epperson C. N. (2014). A randomized, placebo-controlled, double-blind trial of sertraline for postpartum depression. Psychopharmacology 231, 939–948. 10.1007/s00213-013-3316-1 24173623 PMC3945214

[B13] Higgins J. P. T. Altman D. G. Gøtzsche P. C. Jüni P. Moher D. Oxman A. D. (2011). The Cochrane Collaboration’s tool for assessing risk of bias in randomised trials. Bmj Br. Med. J. 343, 889–893. 10.1136/bmj.d5928 PMC3196245 22008217

[B14] Howard L. M. Molyneaux E. Dennis C-L. Rochat T. Stein A. Milgrom J. (2014). Non-psychotic mental disorders in the perinatal period. Lancet 384, 1775–1788. 10.1016/S0140-6736(14)61276-9 25455248

[B15] Hutton B. Salanti G. Caldwell D. M. Chaimani A. Schmid C. H. Cameron C. (2015). The PRISMA extension statement for reporting of systematic reviews incorporating network meta-analyses of health care interventions: Checklist and explanations. Ann. Intern. Med. 162, 777–784. 10.7326/M14-2385 26030634

[B16] Kanes S. Colquhoun H. Gunduz-Bruce H. Raines S. Arnold R. Schacterle A. (2017). Brexanolone (SAGE-547 injection) in post-partum depression: A randomised controlled trial. Lancet 390, 480–489. 10.1016/S0140-6736(17)31264-3 28619476

[B17] Kashani L. Eslatmanesh S. Saedi N. Niroomand N. Ebrahimi M. Hosseinian M. (2017). Comparison of saffron versus fluoxetine in treatment of mild to moderate postpartum depression: A double-blind, randomized clinical trial. Pharmacopsychiatry 50, 64–68. 10.1055/s-0042-115306 27595298

[B18] Ko J. Y. Rockhill K. M. Tong V. T. Morrow B. Farr S. L. (20042008 2017). Trends in postpartum depressive symptoms - 27 States, 2004, 2008, and 2012.and 2012. MMWR. Morb. Mortal. Wkly. Rep. 66, 153–158. 10.15585/mmwr.mm6606a1 PMC5657855 28207685

[B19] Lam R. W. Parikh S. V. Ramasubbu R. Michalak E. E. Tam E. M. Axler A. (2013). Effects of combined pharmacotherapy and psychotherapy for improving work functioning in major depressive disorder. Br. J. Psychiatry 203, 358–365. 10.1192/bjp.bp.112.125237 24029535

[B20] Li H. J. Martinez P. E. Li X. Schenkel L. A. Nieman L. K. Rubinow D. R. (2020). Transdermal estradiol for postpartum depression: Results from a pilot randomized, double-blind, placebo-controlled study. Arch. Womens Ment. Health 23, 401–412. 10.1007/s00737-019-00991-3 31372757 PMC10105981

[B21] Maguire J. Mody I. (2008). GABA(A)R plasticity during pregnancy: Relevance to postpartum depression. Neuron 59, 207–213. 10.1016/j.neuron.2008.06.019 18667149 PMC2875248

[B22] Meltzer-Brody S. Colquhoun H. Riesenberg R. Epperson C. N. Deligiannidis K. M. Rubinow D. R. (2018). Brexanolone injection in post-partum depression: Two multicentre, double-blind, randomised, placebo-controlled, phase 3 trials. Lancet 392, 1058–1070. 10.1016/S0140-6736(18)31551-4 30177236

[B23] Meltzer-Brody S. Howard L. M. Bergink V. Vigod S. Jones I. Munk-Olsen T. (2018). Postpartum psychiatric disorders. Nat. Rev. Dis. Prim. 4, 18022. 10.1038/nrdp.2018.22 29695824

[B24] Mody I. Maguire J. (2011). The reciprocal regulation of stress hormones and GABA(A) receptors. Front. Cell. Neurosci. 6, 4. 10.3389/fncel.2012.00004 22319473 PMC3268361

[B25] Montgomery S. A. Åsberg M. (1979). A new depression scale designed to be sensitive to change. Br. J. Psychiatry 134, 382–389. 10.1192/bjp.134.4.382 444788

[B26] Netsi E. Pearson R. M. Murray L. Cooper P. Craske M. G. Stein A. (2018). Association of persistent and severe postnatal depression with child outcomes. JAMA Psychiatry 75, 247–253. 10.1001/jamapsychiatry.2017.4363 29387878 PMC5885957

[B27] O’Hara M. W. McCabe J. E. (2013). Postpartum depression: Current status and future directions. Annu. Rev. Clin. Psychol. 9, 379–407. 10.1146/annurev-clinpsy-050212-185612 23394227

[B28] Pearson R. M. Evans J. Kounali D. Lewis G. Heron J. Ramchandani P. G. (2013). Maternal depression during pregnancy and the postnatal period: Risks and possible mechanisms for offspring depression at age 18 years. JAMA Psychiatry 70, 1312–1319. 10.1001/jamapsychiatry.2013.2163 24108418 PMC3930009

[B29] Salanti G. Ades A. E. Ioannidis J. P. A. (2011). Graphical methods and numerical summaries for presenting results from multiple-treatment meta-analysis: An overview and tutorial. J. Clin. Epidemiol. 64, 163–171. 10.1016/j.jclinepi.2010.03.016 20688472

[B30] Salanti G. Higgins J. P. T. Ades A. E. Ioannidis J. P. A. (2008). Evaluation of networks of randomized trials. Stat. Methods Med. Res. 17, 279–301. 10.1177/0962280207080643 17925316

[B31] Salanti G. (2012). Indirect and mixed-treatment comparison, network, or multiple-treatments meta-analysis: Many names, many benefits, many concerns for the next generation evidence synthesis tool. Res. Synth. Methods 3, 80–97. 10.1002/jrsm.1037 26062083

[B32] Stewart D. E. Vigod S. (2016). Postpartum depression. N. Engl. J. Med. 375, 2177–2186. 10.1056/NEJMcp1607649 27959754

[B33] Valla L. Wentzel-Larsen T. Smith L. Birkeland M. S. Slinning K. (2016). Association between maternal postnatal depressive symptoms and infants’ communication skills: A longitudinal study. Infant Behav. Dev. 45, 83–90. 10.1016/j.infbeh.2016.10.001 27744111

[B34] Verkuijl N. E. Richter L. Norris S. A. Stein A. Avan B. Ramchandani P. G. (2014). Postnatal depressive symptoms and child psychological development at 10 years: A prospective study of longitudinal data from the South African birth to twenty cohort. Lancet. Psychiatry 1, 454–460. 10.1016/S2215-0366(14)70361-X 26361200

[B35] Vliegen N. Casalin S. Luyten P. (2014). The course of postpartum depression: A review of longitudinal studies. Harv. Rev. Psychiatry 22, 1–22. 10.1097/HRP.0000000000000013 24394219

[B36] Who World health organization. https://www.who.int/clinical-trials-registry-platform (Accessed June 22, 2021).

[B37] Wisner K. L. Hanusa B. H. Perel J. M. Peindl K. S. Piontek C. M. Sit D. K. Y. (2006). Postpartum depression: A randomized trial of sertraline versus nortriptyline. J. Clin. Psychopharmacol. 26, 353–360. 10.1097/01.jcp.0000227706.56870.dd 16855451

[B38] Wisner K. L. Sit D. K. Y. McShea M. C. Rizzo D. M. Zoretich R. A. Hughes C. L. (2013). Onset timing, thoughts of self-harm, and diagnoses in postpartum women with screen-positive depression findings. JAMA Psychiatry 70, 490–498. 10.1001/jamapsychiatry.2013.87 23487258 PMC4440326

[B39] Yonkers K. A. Lin H. Howell H. B. Heath A. C. Cohen L. S. (2008). Pharmacologic treatment of postpartum women with new-onset major depressive disorder: A randomized controlled trial with paroxetine. J. Clin. Psychiatry 69, 659–665. 10.4088/jcp.v69n0420 18363420 PMC3073141

