# Supplementary figures and images for: Comparative efficacy and acceptability of pharmacotherapies for postpartum depression: A systematic review and network meta-analysis
Source: Front Pharmacol. 2022 Nov 24;13:950004. doi: 10.3389/fphar.2022.950004 (PMC9729529; doi:10.3389/fphar.2022.950004)

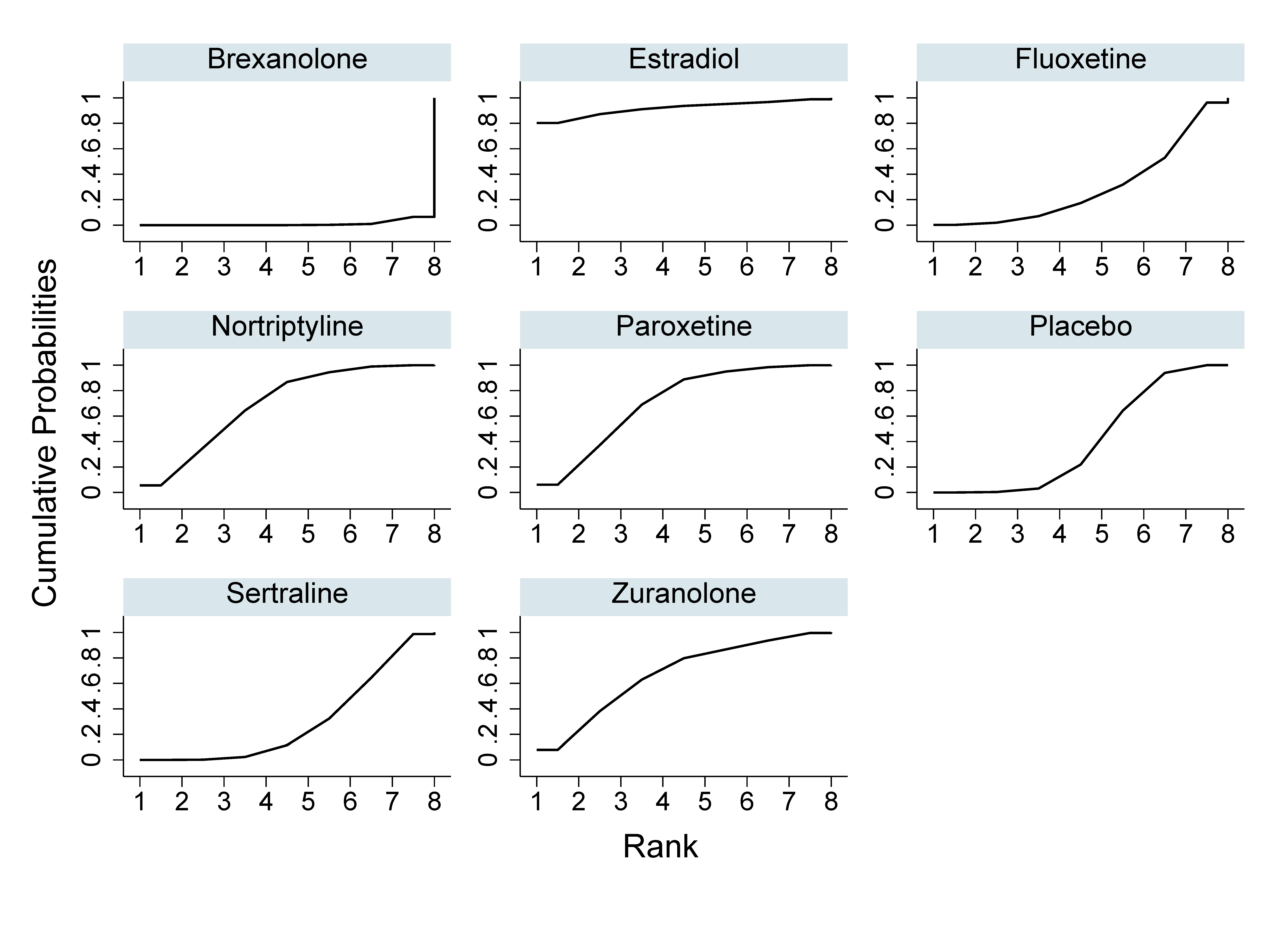

Supplement: Supplementary file 1 [file Image1.TIFF]

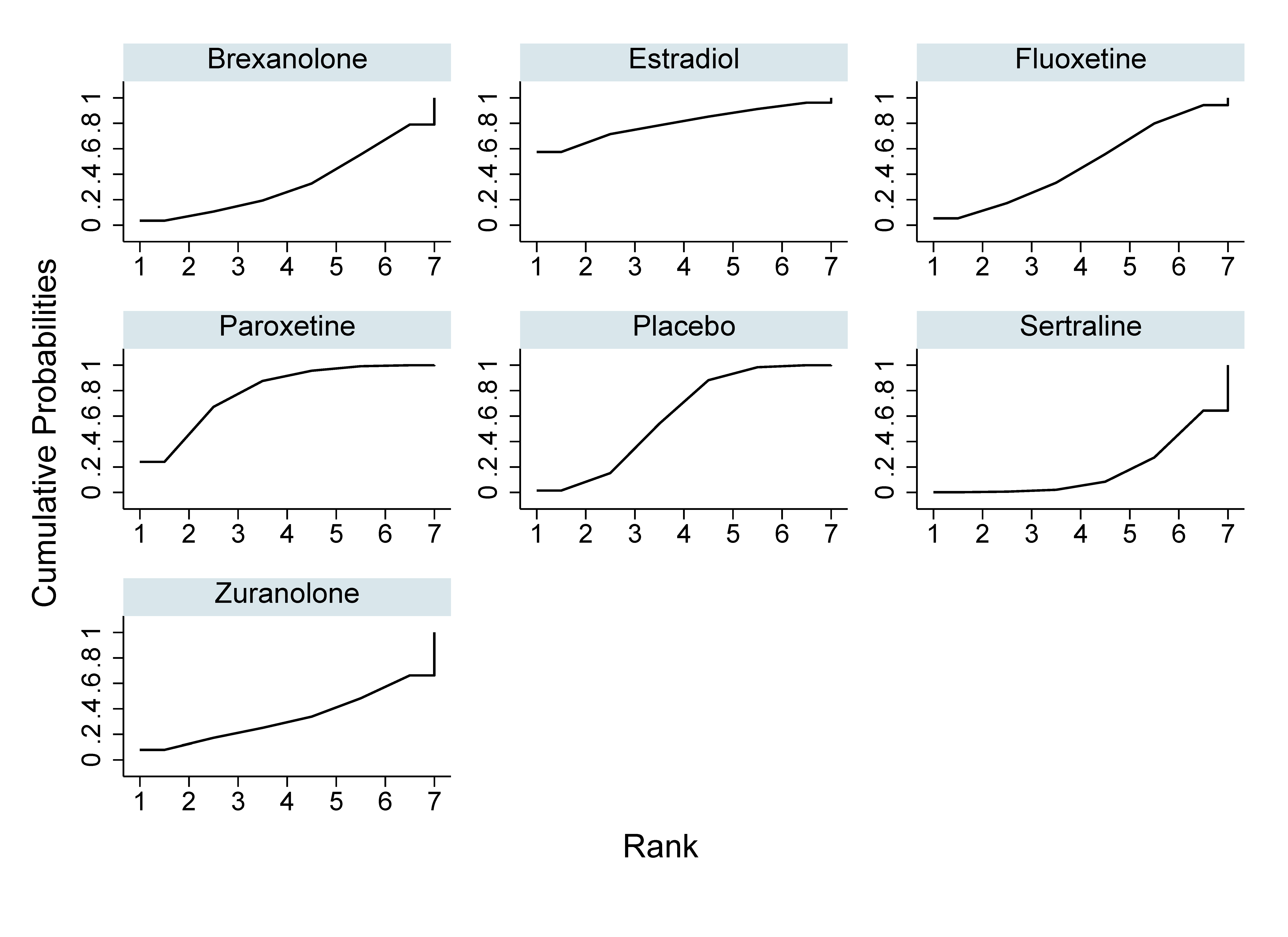

Supplement: Supplementary file 4 [file Image2.TIFF]
