# Supplementary material for: Comparative efficacy and acceptability of pharmacotherapies for postpartum depression: A systematic review and network meta-analysis
Source: Front Pharmacol. 2022 Nov 24;13:950004. doi: 10.3389/fphar.2022.950004 (PMC9729529; doi:10.3389/fphar.2022.950004)
Supplement: Supplementary file 2 [file Table1.docx]

Supplementary Table 1. Network meta-analysis results of the efficacy in terms of MADRS

| **Zuranolone** | 1.16 (-9.30,11.62) | 3.64 (-8.84,16.12) | 5.80 (-3.14,14.74) |
| --- | --- | --- | --- |
| -1.16 (-11.62,9.30) | **Brexanolone** | 2.48 (-7.77,12.73) | 4.64 (-0.78,10.06) |
| -3.64 (-16.12,8.84) | -2.48 (-12.73,7.77) | **Sertraline** | 2.16 (-6.54,10.86) |
| -5.80 (-14.74,3.14) | -4.64 (-10.06,0.78) | -2.16 (-10.86,6.54) | **Placebo** |

**Abbreviations**: MADRS, Montgomery-Åsberg Depression Rating Scale.

**Note**: Network meta-analysis results of the efficacy in terms of standard mean differences for postpartum depression of different antidepressant medications, which are reported in order of surface under the curve cumulative ranking. Top-ranked treatment listed in the top left corner and rankings proceed down the diagonal. lower than 0 favors the column-defining treatment and in the upper right half, those lower than 0 favors the row-defining treatment.
