# Supplementary material for: Comparative efficacy and acceptability of pharmacotherapies for postpartum depression: A systematic review and network meta-analysis
Source: Front Pharmacol. 2022 Nov 24;13:950004. doi: 10.3389/fphar.2022.950004 (PMC9729529; doi:10.3389/fphar.2022.950004)
Supplement: Supplementary file 3 [file Table2.docx]

**Supplementary Table 2. The tolerability profile**

| Study | Treatment | Dropouts for  Any Reason | Dropouts for  Adverse Event | Dropouts for Inefficacy  (Active/PBO) | Serious  Adverse Events  (Active/PBO) | Most Common  Adverse Events in Active Treatment (≥10%) |
| --- | --- | --- | --- | --- | --- | --- |
| Meltzer-Brody-1 | Brexanolone/Placebo | 8/1 | 1/1 | NA | 1/0 | Headache/Dizziness/Somnolence/Infusion site pain/Nausea/Dry mouth/Fatigue |
| Meltzer-Brody-2 | Brexanolone/Placebo | 3/1 | 2/0 | NA | 1/0 | Headache/Dizziness/Somnolence/  Infusion site pain/Nausea/Dry mouth/Fatigue |
| Appleby | Fluoxetine/Placebo | 14/12 | 1/3 | 3/0 | NA | NA |
| O’Hara | Sertraline/Placebo | 24/18 | NA | 2/0 | 10/7 | NA |
| Hantsoo | Sertraline/Placebo | 3/3 | NA | 0/3 | NA | NA |
| Wisner | Sertraline/Nortriptyline | 23/13 | NA | NA | NA | Headache/perspiration  Constipation/Dry mouth/Severe thirst |
| Yonkers | Paroxetine/Placebo | 14/12 | 1/4 | 6/7 | NA | Decreased Appetite/ Diarrhea/Dizziness/Dry Mouth/Headache/Nausea/Somnolence |
| Bloch | Sertraline/Placebo | 2/5 | NA | 2/1 | NA | Hypomanic |
| Deligiannidis | Zuranolone/Placebo | 4/7 | 1/0 | NA | 1/1 | Somnolence/Headache/Dizziness/URTI/Diarrhea/  Sedation/Nausea/Vomiting/Abnormal dreams/  Hyperhidrosis |

**Abbreviations**: NA, not available; URTI, upper respiratory tract infection.
